# Toward standard abbreviations and acronyms for use in articles on aortic disease

**DOI:** 10.1016/j.xjon.2022.04.010

**Published:** 2022-04-20

**Authors:** Zachary G. Perez, Mohammad A. Zafar, Bulat A. Ziganshin, John A. Elefteriades

**Affiliations:** a Aortic Institute at Yale-New Haven Hospital, Yale University School of Medicine, New Haven, Conn; b Department of Cardiovascular and Endovascular Surgery, Kazan State Medical University, Kazan, Russia

**Keywords:** abbreviations, acronyms, aneurysm, aorta, aortic aneurysm

## Abstract

**Objectives:**

Academic medical literature is fraught with complex article-specific acronyms and abbreviations that can impair communication and make reading arduous. Our goal is to ease frustration with bespoke, inconsistent, and variable sets of abbreviations that currently exist for common aorta-related terminology (eg, anatomy, imaging, disease, and therapy). We hope to ease reading and improve communication in the aortic sphere of cardiovascular literature.

**Methods:**

We reviewed a total of 205 published references related to aortic disease, including a systematic review of aorta-related articles in the *Journal of Thoracic and Cardiovascular Surgery* from the years 2020 and 2021. The array of variable definitions, abbreviations, and acronyms encountered in different papers that refer to the same terminology was striking, revealing that there were few standardized abbreviations in the aortic literature. We cataloged these terms, their associated abbreviations, and their frequency of use, and compiled a list of proposed standard abbreviations for commonly used terms that could be implemented uniformly in articles written about aortic diseases.

**Results:**

We present suggested acronyms and abbreviations for common terminology related to the aorta. It is anticipated that this standard list will evolve over time as the literature and technology of the field grows and develops.

**Conclusions:**

A proposed standard set of acronyms and abbreviations for aorta-related terminology is provided that, if found useful, could be implemented broadly in the aortic literature.

## Graphical abstract

Graphical abstract depicting the development and potential influence of a standardized set of abbreviations for aortic terminology.

**Figure:**
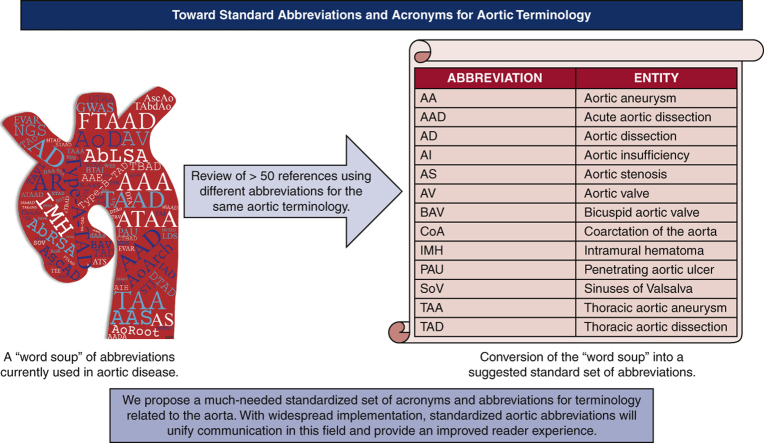

**Figure:**
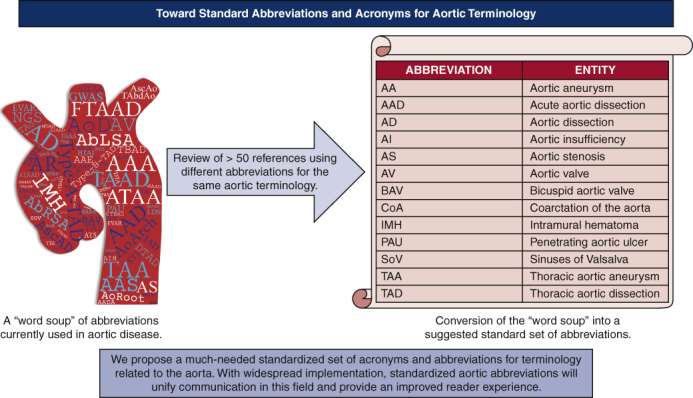
A word soup of abbreviations related to the aorta currently used in the literature.

**Central Message**

We propose a set of standard abbreviations for aortic-related terminology to facilitate better communication and improve the reader experience in the realm of aortic disease.

**Perspective**

Assimilating to complex acronyms and abbreviations at first use in an article makes for a difficult and unsatisfactory reader experience that may lead to miscommunication. Herein, we propose a standard set of abbreviations for aortic-related terminology. Widespread implementation of such abbreviations will likely improve comprehensibility of the literature and enhance the reader's experience.

Among the most onerous aspects of reading scientific research is familiarizing oneself with the plethora of article-specific acronyms and abbreviations. Readers often struggle to remember the exact meaning of each abbreviation in an article at hand, and, need to move up and down the article to find its definition at first use. A combination of increasingly complex research, highly specific terminology, and strict word and character limits is forcing the use of more and more abbreviations. Despite an intention to enhance clarity and ease reading, acronyms and abbreviations can counterproductively become a burden. For many readers, acronyms have contributed to confusion and frustration. In fact, some critics of acronyms and abbreviations have facetiously coined derogatory neologisms for this supposed syndrome.^1^^,^^2^

In the aortic space, many acronyms and abbreviations have arisen to describe anatomic structures, clinical events and syndromes, imaging findings, and surgical and endovascular therapies. In a review of the literature for an upcoming article,^3^, ^4^, ^5^, ^6^, ^7^, ^8^, ^9^, ^10^, ^11^, ^12^, ^13^, ^14^, ^15^, ^16^, ^17^, ^18^, ^19^, ^20^, ^21^, ^22^, ^23^, ^24^, ^25^, ^26^, ^27^, ^28^, ^29^, ^30^, ^31^, ^32^, ^33^, ^34^, ^35^ an overwhelming array of inconsistent abbreviations became apparent.^36^, ^37^, ^38^, ^39^, ^40^, ^41^, ^42^, ^43^, ^44^, ^45^, ^46^, ^47^, ^48^, ^49^, ^50^, ^51^, ^52^, ^53^, ^54^, ^55^, ^56^ After tallying these references, we systematically reviewed all aorta-related articles published in the *Journal of Thoracic and Cardiovascular Surgery* during the years 2020 and 2021. A total of 205 references (inclusive of the initial search and the systematic review) were evaluated. The high degree of variability discovered presents unwanted opportunities for miscommunication between researchers and target audiences as well as foments frustration among readers. It was found that many specific abbreviations had different meanings in different articles. For example, in the literature reviewed, *TAAD* was used as a general term to mean thoracic aortic aneurysms and dissections, as well as a more specific term for both type A aortic dissection and thoraco-abdominal aortic dissection.^12^, ^13^, ^14^^,^^24^ Conversely, there were 13 different acronyms or terms used to denote acute type A thoracic aortic dissection (Table 1).^12^^,^^15^^,^^21^^,^^30^ This finding alone undoubtedly demonstrates that there is a need for an organized method of presenting common aortic terminology in the primary literature.

**Table 1:** Heterogeneity of abbreviations used in reference to type A thoracic aortic dissection in *Journal of Thoracic and Cardiovascular Surgery* articles published during 2020 and 2021

Abbreviation	Entity
AAAD	Acute type A aortic dissection
A-AD	Type A aortic dissection
AAD∗	Acute aortic dissection
AAD∗	Acute type A aortic dissection
AADA	Acute aortic dissection type A
aTAAD∗	Acute type A aortic dissection
ATAAD∗	Acute type A aortic dissection
ATAD	Acute type A dissection
TAAAD	Type A acute aortic dissection
TAAD∗	Type A aortic dissection
TAD∗	Thoracic aortic dissection
TAD∗	Type A dissection
Type A TAD	Type A thoracic aortic dissection

∗ Acronyms that have also been used in the literature to refer to other aortic terminology.

We present a suggested master list of acronyms and abbreviations related to aortic disease that could be used more consistently across journals to achieve both clarity and brevity (Figure 1 and Table 2). Ideally, a single set of standard abbreviations will be used collaboratively among journals within this specialty to minimize the so-called word soup of aortic acronyms that currently exists. Toward this end, we suggest that these acronyms be implemented by all journals that publish frequently on aortic diseases via their Information for Authors document (or the equivalent) to encourage this standardization within the literature. Such an approach would guide authors on what acronyms, if any, to use if they wish to publish on aortic diseases in those journals. This would not replace current conventions to define all acronyms at first use—this is still needed for transparency and clarity. The suggested list can be updated as the literature demands or at regular intervals to reflect new terminologies and therapies. We hope that the widespread implementation of such a list will ease writing, improve communication, and make reading of individual papers less onerous to the reader.

**Figure 1:**
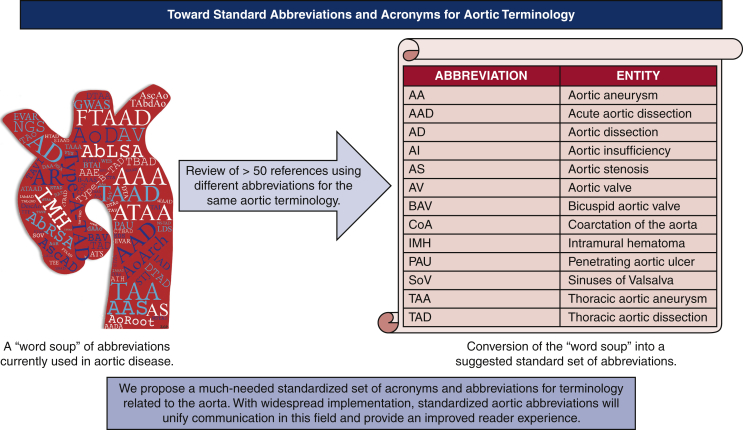
The development and potential influence of a standardized set of abbreviations for aortic terminology. Please see all aorta-related articles in volumes 159 through 162 of *The Journal of Thoracic and Cardiovascular Surgery* and for the remaining articles reviewed for this study.

**Table 2:** Suggested list of standard abbreviations and acronyms for aorta-related terminology

Abbreviation	Entity
AA∗	Aortic aneurysm
AAA	Abdominal aortic aneurysm
AAD	Acute aortic dissection
AAE	Adverse aortic event(s)
AAR	Ascending aortic replacement
AAS	Acute aortic syndrome
ACP	Antegrade cerebral perfusion
AD∗	Aortic dissection
AI	Aortic insufficiency
Ao	Aorta
AoArch	Aortic arch
AoRoot	Aortic root
AR	Aortic regurgitation
ARR	Aortic root replacement
AS	Aortic stenosis
AscAD	Ascending aortic dissection
AscAo	Ascending aorta
ATAA	Ascending thoracic aortic aneurysm
ATS	Arterial tortuosity syndrome
AU	Aortic ulcer
AV	Aortic valve
BArch	Bovine arch
BAV	Bicuspid aortic valve
CA	Celiac artery
CoA	Coarctation of the aorta
CTA	Computed tomography angiography
DescAo	Descending aorta
DHCA	Deep hypothermic circulatory arrest
DTAA	Descending thoracic aortic aneurysm
DTAD	Descending thoracic aortic dissection
DTAo	Descending thoracic aorta
EVAR	Endovascular aneurysm repair
FEVAR	Fenestrated endovascular aneurysm repair
FL	False lumen
HAR	Hemiarch replacement
HCA	Hypothermic circulatory arrest
IA	Innominate artery
IAbdAD	Isolated abdominal aortic dissection
IAD	Iatrogenic aortic dissection
IMA	Inferior mesenteric artery
IMH	Intramural hematoma
LCCA	Left common carotid artery
LRA	Left renal artery
LSCA	Left subclavian artery
LVA	Left vertebral artery
MRA	Magnetic resonance angiography
PAU	Penetrating aortic ulcer
RCCA	Right common carotid artery
RCP	Retrograde cerebral perfusion
RRA	Right renal artery
RSCA	Right subclavian artery
RVA	Right vertebral artery
SAVR	Surgical aortic valve replacement
SMA	Superior mesenteric artery
SoV	Sinuses of Valsalva
STJ	Sinotubular junction
TAA∗	Thoracic aortic aneurysm
TAAA∗	Thoracoabdominal aortic aneurysm
TAAD∗	Thoracic aortic aneurysms and dissections
TAbdAD	Thoracoabdominal aortic dissection
TAbdAo	Thoracoabdominal aorta
TAD∗	Thoracic aortic dissection
TAo	Thoracic aorta
TAR	Total arch replacement
TAV	Tricuspid aortic valve
TAVI	Trans-catheter aortic valve implantation
TAVR	Trans-catheter aortic valve replacement
TEE	Transesophageal echocardiography
TEVAR	Thoracic endovascular aneurysm repair
TL	True lumen
TTE	Transthoracic echocardiography
Type A TAD∗	Type A thoracic aortic dissection
Type B TAD∗	Type B thoracic aortic dissection
VSARR	Valve-sparing aortic root replacement

∗ Preceding term specifiers should be written out unless they are used extensively. These include but are not limited to: acute, chronic, complicated, uncomplicated, sporadic, familial, heritable, iatrogenic, isolated, localized, and spontaneous.

## Conclusions

The suggested list is a starting point toward a communal master list of aortic acronyms and abbreviations. We welcome suggestions for improvement—via alternatives or additions to the entries in this suggested list.

### Conflict of Interest Statement

Dr Elefteriades is the principal of CoolSpine. All other authors report no conflict of interest.

The *Journal* policy requires editors and reviewers to disclose conflicts of interest and to decline handling or reviewing manuscripts for which they have a conflict of interest. The editors and reviewers of this article have no conflicts of interest.
